# Mechanism of proteasome gate modulation by assembly chaperones Pba1 and Pba2

**DOI:** 10.1016/j.jbc.2022.101906

**Published:** 2022-04-06

**Authors:** Helena M. Schnell, Jessie Ang, Shaun Rawson, Richard M. Walsh, Yagmur Micoogullari, John Hanna

**Affiliations:** 1 Department of Pathology, Harvard Medical School and Brigham and Women's Hospital, Boston, Massachusetts, USA; 2 Harvard Cryo-Electron Microscopy Center for Structural Biology, Harvard Medical School, Boston, Massachusetts, USA; 3 Department of Biological Chemistry and Molecular Pharmacology, Blavatnik Institute, Harvard Medical School, Boston, Massachusetts, USA

**Keywords:** proteasome, gate, Pba1, Pba2, HbYX, core particle, Cas9, CRISPR-associated protein 9, CP, core particle, HbYX, hydrophobic-tyrosine-any amino acid, RP, regulatory particle

## Abstract

The active sites of the proteasome are housed within its central core particle (CP), a barrel-shaped chamber of four stacked heptameric rings, and access of substrates to the CP interior is mediated by gates at either axial end. These gates are constitutively closed and may be opened by the regulatory particle (RP), which binds the CP and facilitates substrate degradation. We recently showed that the heterodimeric CP assembly chaperones Pba1/2 also mediate gate opening through an unexpected structural arrangement that facilitates the insertion of the N terminus of Pba1 into the CP interior; however, the full mechanism of Pba1/2-mediated gate opening is unclear. Here, we report a detailed analysis of CP gate modulation by Pba1/2. The clustering of key residues at the interface between neighboring α-subunits is a critical feature of RP-mediated gate opening, and we find that Pba1/2 recapitulate this strategy. Unlike RP, which inserts at six α-subunit interfaces, Pba1/2 insert at only two α-subunit interfaces. Nevertheless, Pba1/2 are able to regulate six of the seven interfacial clusters, largely through direct interactions. The N terminus of Pba1 also physically interacts with the center of the gate, disrupting the intersubunit contacts that maintain the closed state. This novel mechanism of gate modulation appears to be unique to Pba1/2 and therefore likely occurs only during proteasome assembly. Our data suggest that release of Pba1/2 at the conclusion of assembly is what allows the nascent CP to assume its mature gate conformation, which is primarily closed, until activated by RP.

A key feature of protein degradation by the proteasome and related proteases is the sequestration of their proteolytic active sites within an enclosed chamber. In the case of the eukaryotic proteasome, this chamber is the barrel-shaped 20S core particle (CP), which consists of four stacked heptameric rings. The inner β-rings harbor the three distinct proteolytic active sites. Access of substrates to the CP interior is mediated by the outer α-rings, which contain a gate at either axial end. That gate, however, exists primarily in a closed state and requires an activating mechanism of gate opening to facilitate degradation. The primary CP activator is the 19S regulatory particle (RP), an ∼900 kDa multisubunit complex, which binds to the outer surface of the α-rings to create the full proteasome holoenzyme. In addition to recognizing ubiquitinated substrates, deubiquitinating them, and unfolding them, the RP also opens the CP gate and threads the substrate into the CP interior for proteolysis (1).

The RP can be further divided into lid and base subcomplexes (2). The base contains a heterohexameric ring of AAA-type ATPases known as Rpt1–6, which directly bind to the CP surface. Three of these subunits possess a C-terminal HbYX (hydrophobic-tyrosine-any amino acid) motif (3, 4) which inserts into pockets between neighboring α-subunits with a key-in-lock mechanism. The insertion of both the HbYX-containing and non-HbYX-containing subunits (especially Rpt1 and Rpt6) aligns the RP–CP interface and leads to opening of the CP gate (5, 6). A second HbYX-containing CP modulator, the ∼250 kDa monomeric protein Blm10 (PA200 in mammals), also binds to the axial surface of the CP and modulates the gate (7). However, the gate in Blm10–CP complexes is largely disordered with at most a partially open conformation, and the precise function of Blm10 remains uncertain. A third CP activator, PA28, is absent from yeast. Interestingly, and unlike RP and Blm10, PA28 does not possess the HbYX motif (8).

HbYX motifs are also found in the proteins Pba1 and Pba2, which are CP assembly chaperones that “cap” the CP in a manner analogous to the RP and Blm10 (9, 10). Pba1/2 associate early during the assembly process and are released only upon completion of assembly. Binding of Pba1/2 or RP to the CP appears to be mutually exclusive, and one function of Pba1/2 is thought to be prevention of premature activation of the CP by RP; Pba1/2 are also thought to prevent unproductive α–α ring dimerization (9, 11, 12). The HbYX motifs of Pba1/2 also insert into α-ring pockets and appear to make important interactions with residues that influence gating (13). However, there has been no rationale for an open gate during CP assembly and, in fact, reconstitution of recombinant bacterially produced Pba1/2 with mature yeast CP failed to activate the CP *in vitro* (13). Recent cryo-EM structures of CP assembly intermediates indicate a highly unexpected structural arrangement whereby the N terminus of Pba1 is inserted through an open gate into the CP interior where it contacts immature elements of the CP, including another assembly chaperone, Ump1 (Fig. 1; (14)). These findings indicate that Pba1/2, similar to the RP, are capable of mediating an open gate conformation; however, the CP pore itself is occluded by the N terminus of Pba1, explaining why Pba1/2 fail to stimulate CP activity *in vitro*.

**Figure 1:**
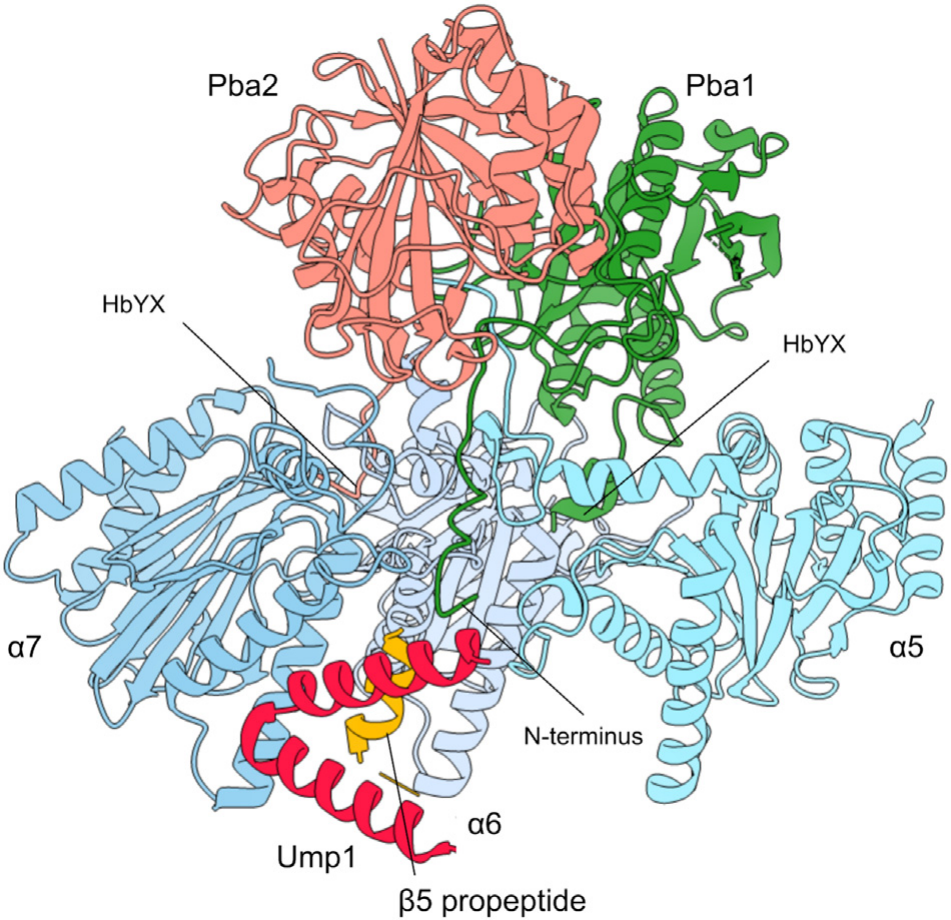
**Structure of Pba1/2 bound to immature CP.** Partial view of the pre-15S assembly intermediate (Protein Data Bank code: 7LS6) showing how the N terminus of Pba1 transits through an open CP pore to contact Ump1 and the β5 propeptide. The C termini of Pba1 and Pba2 are inserted into the α5/6 and α6/7 interfaces, respectively. Note that the final five residues in Pba2 were not resolved in this structure. α1–4 subunits, β2–4 subunits, and parts of Ump1 and β5 are omitted for clarity. CP, core particle.

The process of RP-mediated gate opening is incompletely understood, but two important conceptual aspects of gate opening have become apparent. The CP gate is formed by the N termini of the α-subunits (15). In the closed state, there is a critical multifaceted interaction between the N termini of α2–4 that accounts for much of the density at the center of the gate. The main residues involved include Phe7 in α2, Asp7 in α3, and Tyr4 and Arg6 in α4 (6, 15). α2-Phe7, in particular, has been proposed to nucleate this cluster of residues (6). Importantly, disruption of this interaction by either mutation or deletion results in constitutively active open-gate CP (15). The second major feature of RP-mediated gate opening concerns a highly unique clustering of residues at each α–α subunit interface (8, 16). In each case, a Tyr–Asp–Pro–Tyr tetrad clusters at the periphery of the pore through intermolecular interactions that facilitate the open gate conformation. In particular, the proximal tyrosine and aspartate residues, which are centrally located in the closed state, snap back to their more peripheral locations in the open state. Direct interactions between individual Rpt subunits and α-subunits appear to explain how at least some of these tetrad pairing interactions are stimulated by RP binding (6). However, the RP does not make direct interactions within the central α2/3/4 cluster, and so it is unclear (other than by general allosteric considerations) how the RP disrupts this interaction, which is a prerequisite for gate opening.

Here, we have analyzed the mechanism of CP gate modulation by Pba1/2. Like the RP, Pba1/2 also utilize the Tyr–Asp–Pro–Tyr tetrad clustering strategy, supporting the notion that this may be a general principle of gate opening. However, unlike the RP, Pba1 makes extensive contacts within the central aspect of the gate, disrupting the central cluster formed by α2, α3, and α4. Our data indicate a unique two-pronged approach to gate modulation by Pba1/2, which likely exists only during proteasome biogenesis.

## Results

### The CP shows an open gate conformation in CP assembly intermediates

**Figure 2:**
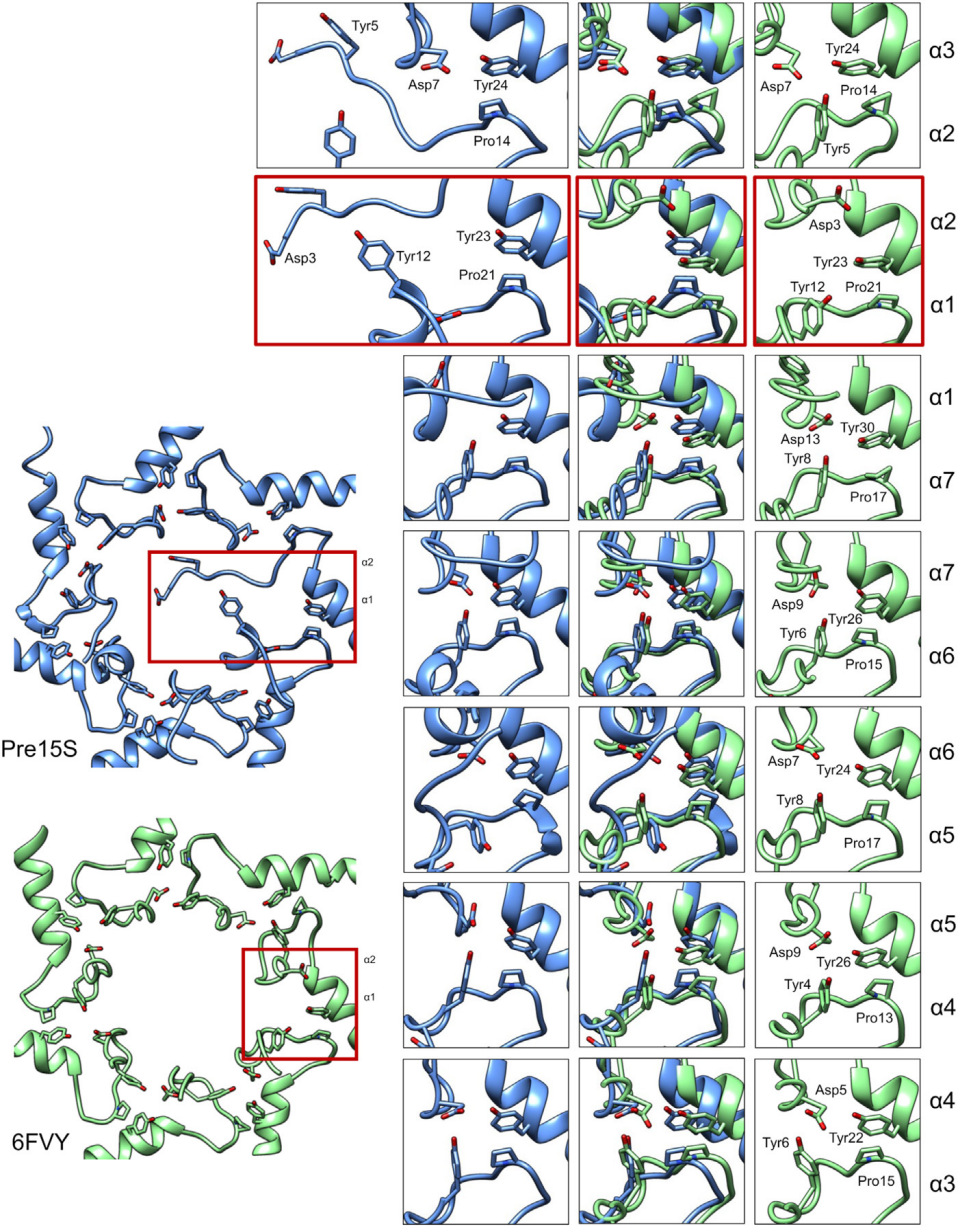
**Open gate conformation in CP assembly intermediates.** Gate conformation of the pre-15S assembly intermediate (Protein Data Bank code: 7LS6) and RP–CP (Protein Data Bank code:6FVY) are shown at *left*. Close-up views of the Tyr–Asp–Pro–Tyr tetrads are shown at *right*. The α1/2 interface is highlighted in *red*. CP, core particle; RP, regulatory particle.

We recently showed that proteasome assembly intermediates bound to Pba1/2 show an essentially open gate conformation (14). A key feature of the open gate conformation in RP-bound CP is the Tyr–Asp–Pro–Tyr tetrad clustering that occurs at the interfaces between neighboring α-subunits. For example, at the α3/α4 interface, Tyr6 from α3 and Asp5 from α4—both of which would be present at the center of the CP gate in the closed state—are retracted to a more peripheral location where they pair with each other and with the α3-Pro15/α4-Tyr22 pair (Fig. 2, *right-most panels*). Analogous interactions occur at all seven subunit interfaces within the RP-activated CP (Fig. 2).

We sought to determine whether Pba1/2 pursued a similar tetrad clustering strategy as the RP. We found that the respective tetrad clusters were engaged in a manner nearly superimposable on RP-activated CP for four of the seven subunit pairs (Fig. 2). The exceptions were α7/α1, α1/α2, and α2/α3. In contrast to RP-activated CP, the N terminus of α2 was not retracted but rather showed an extended conformation that descended into the interior of the CP, as we previously described (14). In addition, a portion of the N terminus of α1, including the critical Tyr12 residue, was also in an extended state, although less dramatically so (Fig. 2). The α7/α1 cluster was disrupted *via* movement of α1-Asp13 (see later). At all three interfaces, the distal Pro–Tyr pairs were maintained, but the Tyr–Asp interactions were disrupted. These results suggest that binding of Pba1/2 recapitulates key aspects of RP-mediated gate opening and suggests that such tetrad pairing may be a generalizable feature of CP gate modulation.

### Direct interactions between Pba1/2 and Tyr–Asp–Pro–Tyr tetrad clusters

Pba1 and Pba2 directly interact with six of the seven tetrad clusters, explaining how these chaperones achieve the open gate conformation during CP assembly.

#### Alpha4–Alpha5

**Figure 3:**
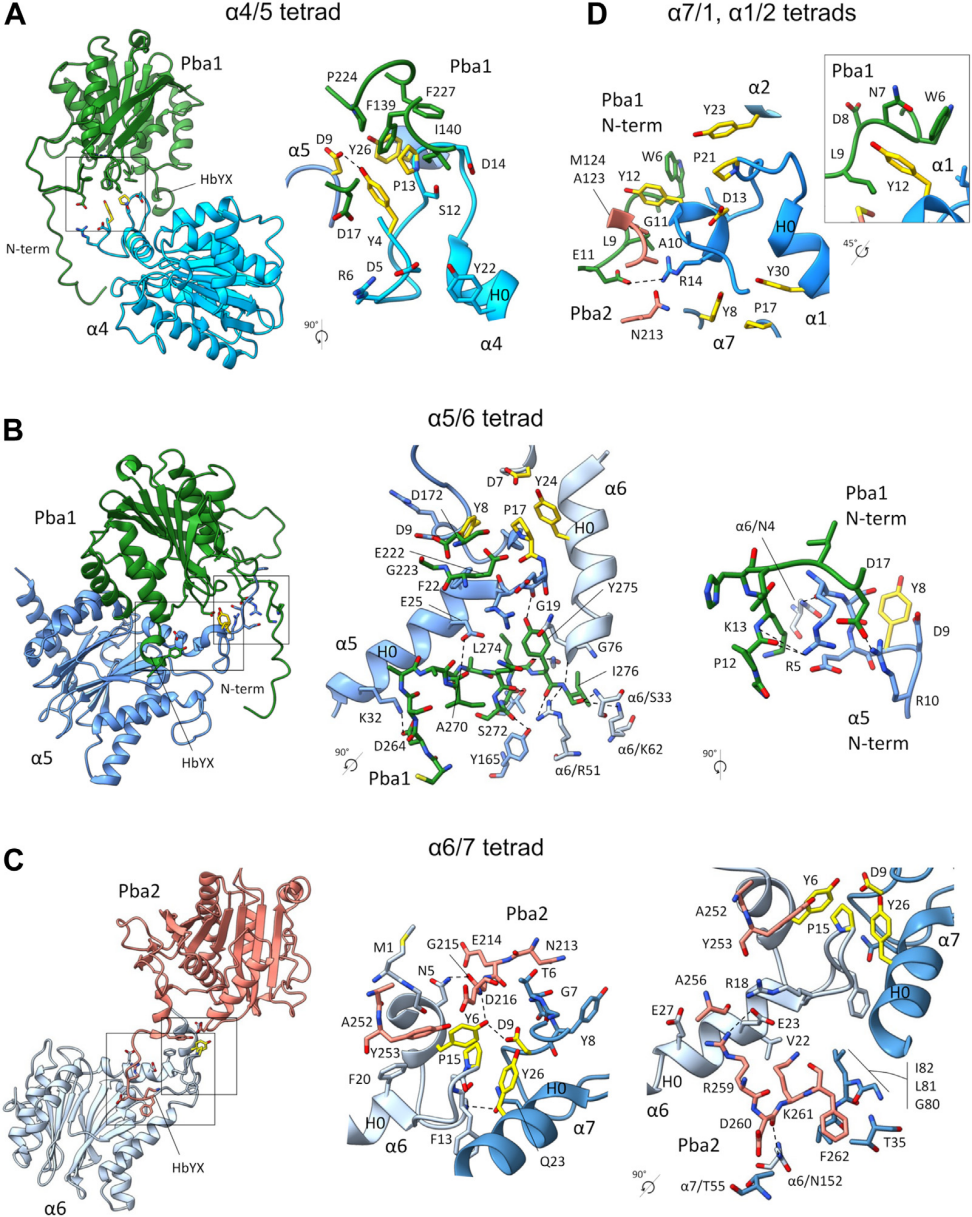
**Interactions between Pba1/2 and Tyr–Asp–Pro–Tyr tetrad clusters.***A*, contacts between Pba1 and the α4/α5 tetrad with close-up view of the boxed region shown in the *right panel*. *B*, contacts between Pba1 and the α5/α6 tetrad with close-up views of the boxed regions shown in the *middle* and *right panels*. The *right panel* highlights the interaction of the N terminus of Pba1 with the N terminus of α5. *C*, contacts between Pba2 and the α6/α7 tetrad with close-up views of the tetrad area (*middle panel*) and C terminus of Pba2 (*right panel*). *D*, close-up view of contacts between Pba1 and the α7/α1 and α1/α2 tetrads. The *boxed inset* shows how N-terminal residues 6 to 9 of Pba1 create a binding pocket for α1-Tyr12. In all panels, residues involved in tetrad clustering are colored in *yellow*; hydrogen bonds and salt bridges are indicated by *dashed lines*. Backbone atoms are shown to indicate backbone bonds. Interactions were determined by PDBePISA (31).

A group of Pba1 residues, including Phe139, Phe227, and Ile140, contacts one side of this tetrad to interact with the key Pro13 of α4 (Fig. 3*A* and Table S1). On the other side of the tetrad, Asp17 from the N terminus of Pba1 contacts the key Tyr4 of α4. These interactions appear to support the engagement of this tetrad cluster.

#### Alpha5–Alpha6

Glu222 and D172 in Pba1 are closely associated with the Pro17/Tyr8 pair in α5 (Fig. 3*B*, *left and middle panels*). Furthermore, Tyr275 from the HbYX motif of Pba1 is hydrogen bonded to Gly19 in α5, which likely stabilizes the position of the nearby Pro17. In fact, much of the C terminus of Pba1 is engaged in a complex arrangement of hydrogen bonding and salt bridge interactions with residues from α5 and α6 that appears to precisely position the C terminus of Pba1 within the α5/α6 pocket.

**Figure 4:**
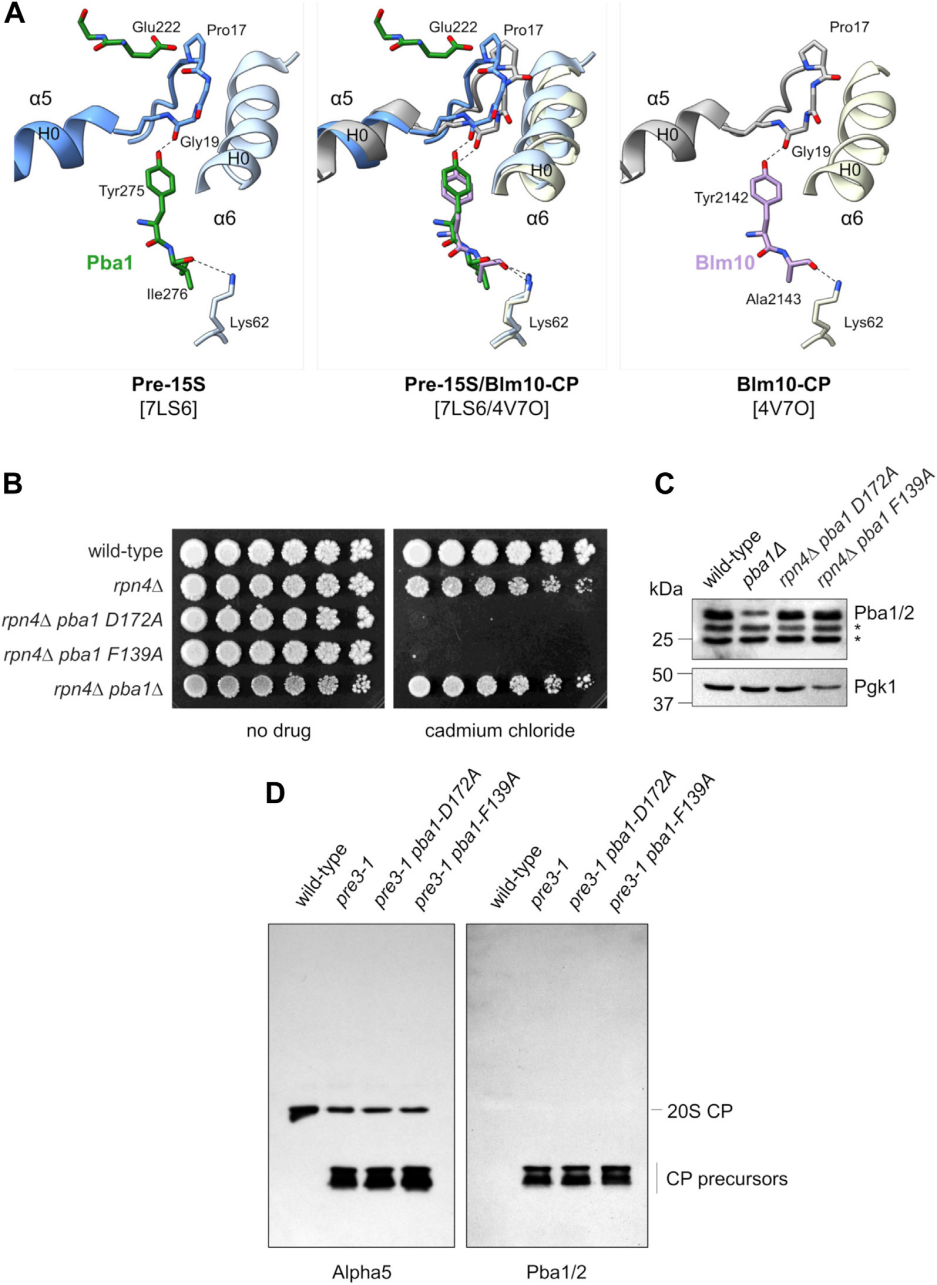
**Further analysis of interactions between Pba1 and Tyr–Asp–Pro–Tyr tetrad clusters**. *A*, interaction of the Pba1 (*left panel*) or Blm10 (*right panel*) HbYX motif with the α5/α6 pocket. In both cases, the penultimate tyrosine residue of Pba1 or Blm10 is hydrogen bonded to α5-Gly19, with the final residue engaged in a salt bridge with α6-Lys62. Backbone atoms are shown to indicate backbone carbonyl bonds. *B*, growth defect of Pba1 mutants involved in α-subunit tetrad clustering. Pba1–Asp172 contributes to α5/α6 clustering (Fig. 3*B*). Pba1–F139 contributes to α4/α5 clustering Fig. 3*A*). Cells were spotted in threefold serial dilutions onto plates lacking or containing cadmium chloride (30 μM) and cultured for 3 to 5 days. Similar results were obtained in two independent experiments. *C*, Pba1/2 protein levels in the indicated mutants. Whole-cell extracts were prepared and analyzed by SDS-PAGE followed by immunoblotting with antibodies to Pba1/2 (*upper panel*) and Pgk1 (*lower panel*). Note that Pba1 and Pba2 are of similar size and comigrate electrophoretically. *Asterisks*, nonspecific bands. Similar results were obtained in two independent experiments. *D*, Pba1–D172A and Pba1–F139A mutants retain binding to the proteasome. Purified CP (1.3 μg) from the indicated strains was analyzed by native gel electrophoresis followed by immunoblotting with the indicated antibodies. The positions of mature 20S CP and sub-20S CP assembly intermediates are indicated. Similar results were obtained in two separate experiments. CP, core particle; HbYX, hydrophobic-tyrosine-any amino acid.

A prior crystal structure of recombinant bacterially produced Pba1/2 bound to mature MG132-inhibited yeast CP has been reported (13). Interestingly, the contacts made by HbYX motif of Pba1 are different from those seen here in that the prior structure shows Pba1–Tyr275 being hydrogen bonded to Leu21 and Glu25 of α5 (13). Nevertheless, the arrangement of sole HbYX motif of Blm10 (7) is remarkably similar to the Pba1 arrangement seen here. For both Blm10 and Pba1, the penultimate Tyr residue is hydrogen bonded to α5-Gly19, while a salt bridge is present between the last residue and α6-Lys62 (Fig. 4*A*).

The N terminus of Pba1 also makes a multifaceted interaction at this site, essentially creating a grooved surface, which wraps around N terminus of α5 (Fig. 3*B*, *right panel*). This interaction is centered around α5-Arg5 and further stabilized through hydrogen bonding between α5 and both Pba1 and α6. This arrangement appears to further promote the positioning of the key Tyr8 of α5 to facilitate tetrad clustering.

#### Alpha6–Alpha7

The interaction of Pba2 at this tetrad is analogous to that of Pba1 with α5/α6. Pba2–Asp216 contacts the Tyr6–Pro15 pair from α6, with a direct hydrogen bond to Tyr6 (Fig. 3*C*, *left and middle panels*). The position of Asp216 appears to be further enforced by its neighbor Gly215, which in turn is hydrogen bonded to α6-Asn5.

At the C terminus, Pba2–Tyr253 further interacts with the tetrad (Fig. 3*C*, *middle panel*), and as in Pba1, the overall position of the C terminus of Pba1 within the α6/α7 pocket also appears to be enforced through a network of hydrogen bonding and salt bridge interactions (Fig. 3*C*, *right panel*).

#### Alpha7–Alpha1 and Alpha1–Alpha2

These two tetrad clusters are partially disrupted. The principal feature accounting for this disruption appears to be the direct interaction between the N terminus of Pba1 and the key Tyr12 residue of α1 (Fig. 3*D*), which directly disrupts the α1/α2 tetrad by pulling Tyr12 away. Pba1 residues 6 to 9 appear to create a binding pocket for α1-Tyr12, with Pba1–Trp6 and Pba1–Leu9 sitting at either end of the pocket (Fig. 3*D*, *inset*). The movement of α1-Tyr12 also appears to pull the neighboring Asp13 residue of α1 away from the rest of the α7/α1 tetrad (Fig. 3*D*).

#### Alpha2–Alpha3

**Figure 5:**
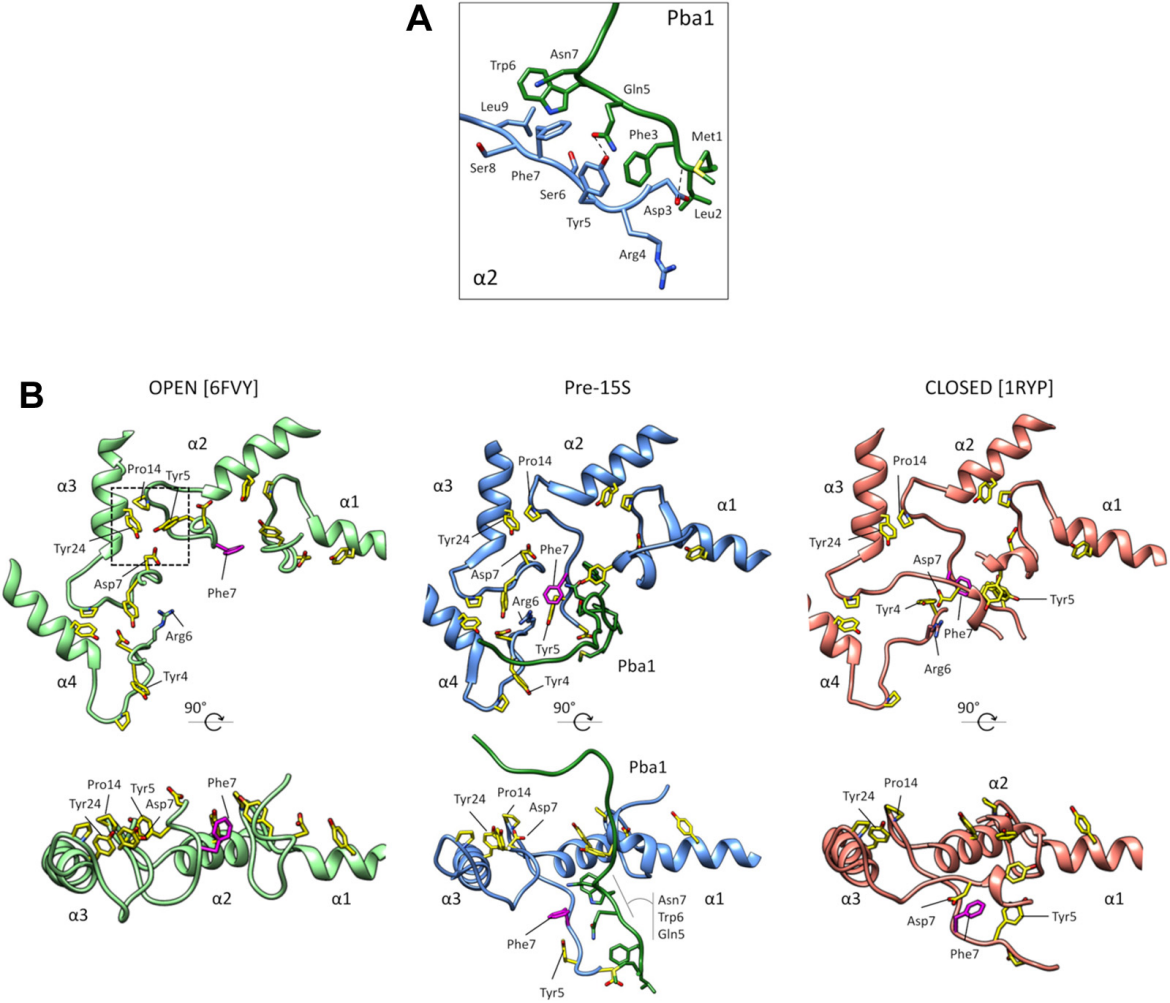
**The N terminus of Pba1 disrupts the alpha2–4 cluster at the center of the gate**. *A*, contacts between the N termini of Pba1 and α2. *B*, partial views of RP-activated CP with an open gate state (Protein Data Bank [PDB] code: 6FVY), Pba1/2 bound to the pre-15S assembly intermediate (PDB code: 7LS6), and free 20S with closed gate (PDB code: 1RYP) conformation. Residues involved in tetrad clusters are colored in *yellow*; α2-Phe7 is colored in *magenta*. CP, core particle; RP, regulatory particle.

The N termini of Pba1 and α2 form an extended interface that is directed toward the CP interior (Fig. 5*A*). This interaction was not visualized in the previous Pba1/2–CP–MG132 crystal structure (13) because of disorder that left the first 27 residues of Pba1 unresolved. The N terminus of Pba1 interacts with the key Tyr5 residue of α2, and Pba1–Gln5 is hydrogen bonded at that site (Fig. 5*A*), explaining why the N terminus of α2 is extended rather than retracted, and why the α2/α3 tetrad is disrupted (Fig. 2). Overall, the interactions just described explain the tetrad clustering outcomes at six of the seven α-subunit interfaces.

We sought to test the physiologic significance of these interactions. The *pba1Δ* mutant alone shows mild phenotypes, but these can be exacerbated by loss of Rpn4 whose role is to stimulate proteasome biogenesis in response to stress (17, 18). We introduced the Pba1–D172A and F139A mutations, which affect the α5/6 and α4/5 tetrads, respectively, into the *rpn4Δ* background by CRISPR-associated protein 9 (Cas9)–mediated gene editing. Each mutation decreased growth upon exposure to cadmium chloride, a divalent heavy metal that causes protein misfolding and proteotoxic stress (Fig. 4*B*). These phenotypes were not because of decreased Pba1/2 levels in cells (Fig. 4*C*). Pba1/2 preferentially bind to immature CP, and so there is very little Pba1/2 bound to CP purified from wildtype cells (Fig. 4*D*). Therefore, to evaluate CP binding, we introduced the Pba1–D172A and Pba1–F139A mutations into a *pre3-1* background, which we previously showed has a strong CP assembly defect with accumulation of sub-20S precursors that contain Pba1/2 (14). Both mutants fully retained their ability to bind immature CP species (Fig. 4*D*), indicating that their phenotypic defects reflect their inability to carry out their normal function within CP assembly. In fact, the phenotypes of the *rpn4Δ pba1*–*D172A* and *rpn4Δ pba1*–*F139A* mutants were actually stronger than the *rpn4Δ pba1Δ* mutant under these conditions (Fig. 4*C*).

### N terminus of Pba1 disrupts the alpha2–4 cluster at the center of the gate

Four key residues form a cluster at the center of the closed CP gate: Phe7 in α2, Asp7 in α3, and Tyr4 and Arg6 in α4 (Fig. 5*B*, *right panel*). It has been suggested that α2-Phe7 is the main nucleator or “linchpin” of this cluster (6). Remarkably, the N terminus of Pba1 directly interacts with Phe7 at the center of the gate (Fig. 5, *A* and *B*, *middle panel*), releasing α3-Asp7, α4-Tyr4, and α4-Arg6, all of which are thereby allowed to take positions more typical of the open gate state (Fig. 5*B*). Indeed, the key Tyr4 in α4 is engaged in complete tetrad pairing in the α4–α5 cluster in a manner that is nearly indistinguishable from the open state of RP-activated CP (Fig. 2). Thus, Pba1 directly disrupts the closed-gate cluster between α2, α3, and α4 through its interactions with α2, and it does this by competing with other α-subunits for binding to the N terminus of α2.

**Figure 6:**
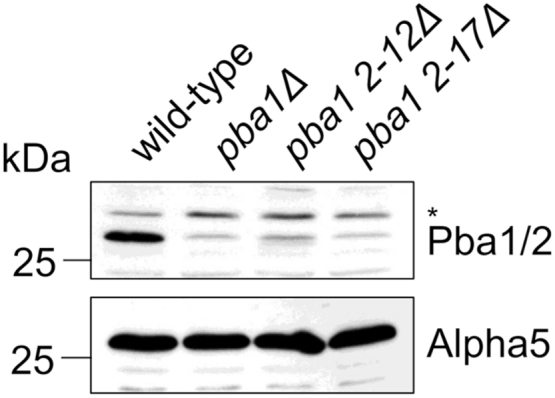
**The N terminus of Pba1 is critical for overall Pba1/2 stability.** Whole-cell extracts were prepared from wildtype, *pba1Δ*, *pba1 2–12Δ*, and *pba1 2–17Δ* cells and analyzed by SDS-PAGE followed by immunoblotting. *Asterisk*, nonspecific band. Similar results were obtained in two independent experiments.

Given the extensive interactions made by the N terminus of Pba1, we sought to determine the consequences of its loss. We used Cas9-mediated gene editing to delete residues 2 to 17 from Pba1. However, upon examination of whole-cell extracts, we found that the Pba1^2–17Δ^ mutant resulted in complete loss of expression of both Pba1 and Pba2 (Fig. 6). We prepared a shorter deletion, Pba1^2–12Δ^, but this also resulted in loss of expression of Pba1 and Pba2 (Fig. 6). The poor expression of the Pba1^2–12Δ^ mutant was surprising since we had previously been able to express this mutant in bacteria and assemble it with Pba2 onto mature yeast CP (14). While these results precluded further analysis of these mutants, they nevertheless indicate that the N terminus of Pba1 plays a critical role in the overall stability or expression of the Pba1/2 dimer.

### Evolutionary conservation of the key residues in Pba1

**Figure 7:**
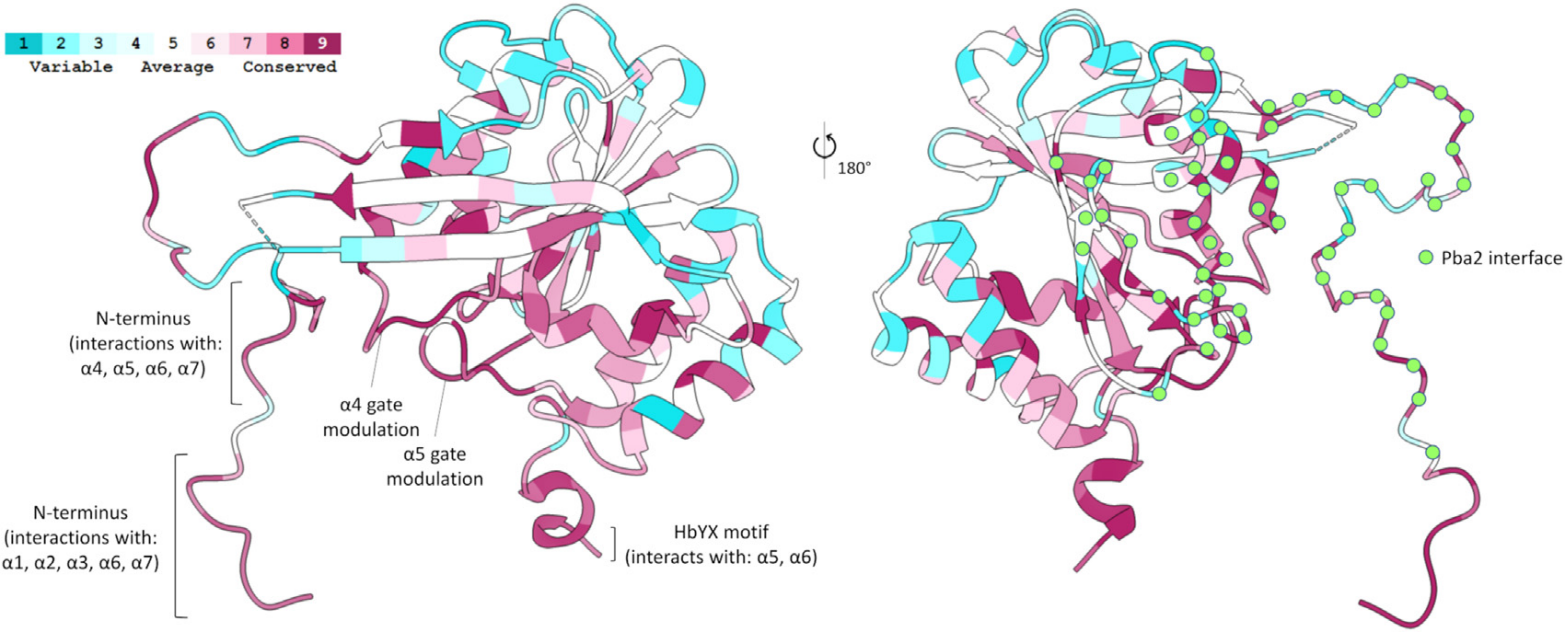
**Evolutionary conservation of the key residues in Pba1**. Structure of Pba1 with residues colored according to evolutionary conservation as determined by ConSurf (28, 29). Residues interacting with Pba2 are marked with *green dots*.

Finally, we sought to evaluate the physiologic significance of these described Pba1 functions by examining the evolutionary sequence conservation of Pba1. Strikingly, a large fraction of its highly conserved residues could be assigned to the regions of Pba1 that mediate gate modulation, namely, its very N terminus, its C-terminal HbYX region, and the areas that contact the tetrad clusters in α4 and α5 (Fig. 7 and Table S2); however, it should be noted that this analysis was limited to lower eukaryotes. Most of the remaining highly conserved residues appeared to either mediate Pba1/2 dimerization or mediate intramolecular interactions within Pba1 that likely facilitate the overall structure of the protein.

## Discussion

### Pba1/2 utilize multiple mechanisms for CP gate modulation

Our recent structures of CP assembly intermediates revealed a highly unexpected structural arrangement whereby the N terminus of Pba1 is inserted through an open gate into the CP interior where it contacts multiple α-subunits as well as immature aspects of the CP (14). Despite the essentially open gate conformation, the outcome of Pba1/2 binding is quite different from that of RP since the pore itself is occluded by the N terminus of Pba1. Here, we have analyzed the detailed mechanisms of CP gate opening by Pba1/2 and have uncovered four new aspects of their function. First, Pba1/2 open the gate through a tetrad clustering strategy similar to the RP, but which differs in its detailed interactions. This approach is also used by Blm10 and PA28, supporting the notion that this may be a general mechanism of gate modulation. Second, we find that Pba1/2 directly modulate six of the seven tetrad clusters. This is remarkable because Pba1/2 are small and insert at only two α-subunit interfaces, in contrast to the RP, which has six subunits (Rpt1–6) inserted into the α-ring. Our results indicate that the gate modulatory activity of Pba1/2 extends well beyond its HbYX motifs as residues distributed throughout both proteins contribute to these interactions. Third, the N terminus of Pba1 plays an important role in some of these tetrad-modulating events. These interactions are especially important on the α1–3 side of the ring since the bulk of Pba1/2 sit on the α5–7 side. Finally, we find that the N terminus of Pba1 directly disrupts the tight cluster of residues from α2, α3, and α4 that sit at the center of the gate. The N terminus of Pba1 appears to achieve this feat by substituting for inter-α-subunit interactions that would normally maintain the gate in its closed state. The RP is not known to contact this site, and therefore, how RP disrupts this tight interaction remains unknown, other than likely being allosteric in nature. We suspect that this mechanism works in concert with the tetrad clustering to achieve the maximal effect on gate modulation; however, we should note that direct binding to the central α2/3/4 cluster is not strictly necessary for gate opening, at least for small peptides *in vitro*, as a Pba1^2–12Δ^/Pba2 mutant was capable of activating mature assembled CP for peptide hydrolysis (14).

The Pba1 gate-modulating interactions we have described are all characterized by a high degree of evolutionary conservation, at least among lower eukaryotes. PAC1 and PAC2, the mammalian orthologs of Pba1 and Pba2, are thought to function similarly to their yeast counterparts but show only limited sequence conservation (9, 10), and no structural information on their interactions with maturing CP is available. Therefore, an important goal for future work will be to determine whether PAC1/2 utilize similar mechanisms of gate modulation.

### Role of the N terminus of Pba1 in CP assembly

Our data indicate that the N terminus of Pba1 contributes to both aspects of gate opening just described. The N terminus of Pba1 also appears to contribute to the role of Pba1/2 in properly configuring the α-ring. During CP assembly, a complete α-ring is formed first, and then the β-subunits are sequentially incorporated (19). However, the precise sequence of events that mediates α-ring assembly remains poorly understood. Pba1/2 orchestrate α-ring assembly with the help of a second heterodimeric chaperone pair, Pba3/4 (20, 21, 22). Interestingly, while Pba3/4 bind to the opposite surface of the α-ring, both Pba1/2 and Pba3/4 are largely located on the α4–7 side of the ring (13, 23). Thus, there has been some uncertainty as to how the remaining subunits, especially α2 and α3, are properly incorporated. Our finding that the N terminus of Pba1 contacts the remaining subunits, with especially strong interactions with α2 and α1, begin to explain this issue. An intriguing possibility would be that Pba1/2 and Pba3/4 bring together opposite sides of the ring, with Pba3/4 bound to the α4–7 side and Pba1/2 bound to the α1–3 side (largely through the N-terminal interactions of Pba1). However, despite its explanatory appeal, little evidence is currently available to support this model. In fact, the main α-ring precursor that has been isolated to date consists of α4–7 bound to both Pba3/4 and Pba1/2, although this precursor was not detected in yeast but rather in mammalian cells and only after knockdown of α1, α2, or α3 (11).

A separate but related issue concerns the dramatic loss of expression of both Pba1 and Pba2 upon deletion of the N terminus of Pba1. We do not fully understand the basis for this observation, but it speaks to the critical nature of this part of Pba1. A complete Pba1 null mutant destabilizes Pba2, and *vice versa*, suggesting that interactions between the proteins are critical for their joint stability (12). Consistent with this finding, Pba1 is insoluble when expressed in bacteria unless Pba2 is coexpressed (10). Much of the N terminus of Pba1 from residue 9 onward interacts with Pba2 (Fig. 7). Therefore, it is possible that the *pba1*^*2–12Δ*^ and *pba1*^*2–17Δ*^ mutants destabilize Pba1 and Pba2 through disruption of their interaction; although, this would make these residues (especially residues 9–12) essentially obligatory for Pba1/2 dimerization despite the fact that the two proteins interact at many other sites. Structural information on free Pba1/2 is not available, but one possibility is that the N terminus of Pba1 might be more extensively bound to Pba2 until Pba1/2 bind to the nascent proteasome. The destabilization of Pba1/2 upon deletion of the N terminus of Pba1 precludes definitive experimental testing of the model that the N terminus of Pba1 makes important contributions to CP biogenesis. Nevertheless, that model is consistent with the extensive interactions made by the N terminus of Pba1 within the α-ring, as shown here, as well as with the assembly chaperone Ump1 and the immature β5-propeptide (see also Ref. (14)).

### Multiple mechanisms of CP gate modulation by proteasome regulators

The major characterized regulators of the CP gate in yeast are the RP, Blm10, and Pba1/2, all of which perform very different functions. The RP mediates gate opening to facilitate protein degradation, whereas Pba1/2 facilitate CP biogenesis. The overall function of Blm10 remains controversial, and opposing views exist on whether it represents a true CP activator *in vivo* as well as its precise effect on gate conformation (7, 24). While the three complexes share some common mechanisms of gate modulation, including the Tyr–Pro–Asp–Tyr tetrad clustering, Pba1/2 appear unique in their ability to directly interact with the cluster of residues from α2–4 that comprise the central aspect of the gate and that are required to maintain the closed state. This interaction, which is without precedent in proteasome biology, implies that the maturing CP cannot assume its final closed gate conformation until the release of Pba1/2 at the end of assembly. The CP gate then presumably remains in this closed state until activated by the RP.

## Experimental procedures

### Strains and antibodies

Yeast strains are listed in Table 1. Point mutations and deletions were introduced using Cas9-mediated gene editing as previously described (25). Plasmids and primers used for those constructions are listed in Tables 2 and 3, respectively. Proteasome affinity tags were introduced by standard homologous recombination–based methods. Mutant strains were validated by targeted sequencing of their genomic DNA. Cells were cultured at 30 °C in yeast extract–peptone–dextrose medium (1% yeast extract, 2% Bacto peptone, and 2% dextrose).

**Table 1:** Yeast strains

Name	Genotype	Source
sJH92	*MAT****a****his3Δ1 leu2Δ0 met15Δ0 ura3Δ0*	RGC
sJA181	*MAT****a****his3Δ1 leu2Δ0 met15Δ0 ura3Δ0 pba1::pba1 2–12Δ*	This study
sJA182	*MAT****a****his3Δ1 leu2Δ0 met15Δ0 ura3Δ0 pba1::pba1 2–17Δ*	This study
sJA208	*MAT****a****his3Δ1 leu2Δ0 met15Δ0 ura3Δ0 rpn4::KAN pba1::pba1-D172A*	This study
sJA209	*MAT****a****his3Δ1 leu2Δ0 met15Δ0 ura3Δ0 rpn4::KAN pba1::pba1-F139A*	This study
sMB163	*MAT****a****ura3 leu2-3, 112 his3-11,15 Can*^*s*^*Gal*^*+*^*PRE1::PRE1-TEV-ProA (HIS3)*	(14)
sMB186	*MAT****a****ura3 leu2-3, 112 his3-11,15 Can*^*s*^*Gal*^*+*^*pre3-1 PRE1::PRE1-TEV-ProA (HIS3)*	(14)
sJA216	*MAT****a****ura3 leu2-3, 112 his3-11,15 Can*^*s*^*Gal*^*+*^*pre3-1 pba1::pba1-D172A PRE1::PRE1-TEV-ProA (HIS3)*	This study
sJA218	*MAT****a****ura3 leu2-3, 112 his3-11,15 Can*^*s*^*Gal*^*+*^*pre3-1 pba1::pba1-F139A PRE1::PRE1-TEV-ProA (HIS3)*	This study
sJA217	*MAT****a****his3Δ1 leu2Δ0 met15Δ0 ura3Δ0 pba1::KAN rpn4::NAT*	This study

Abbreviation: RGC, Research Genetics Collection (available from Thermo Fisher Scientific).

**Table 2:** Yeast plasmids

Name	Description	Source
bRA90	pPGK1 CAS9 (LEU2)	(25)
pJH395	pPGK1 CAS9 + gRNA PBA1 2–12Δ and 2–17Δ (LEU2)	This study
pJH409	pPGK1 CAS9 + gRNA PBA1 F139A (LEU2)	This study
pJH410	pPGK1 CAS9 + gRNA PBA1 D172A (LEU2)	This study

Abbreviation: gRNA, guide RNA.

**Table 3:** Sequences of gRNAs and the Cas9-repair templates used for Cas9-mediated gene editing

Name	Sequence 5′-3′
JA167 PBA1 (gRNA F)	CATATTTTGCTAGAGTAGAGgtttt
JA168 PBA1 (gRNA R)	CTCTACTCTAGCAAAATATGgatca
JA178 PBA1 2-12Δ (Cas9 repair)	ATATCATCGCACTACAGTAAAATTTTCATTTATAGCGATGAAACATCTGCTAGATCTCCCAGAGATTTCAAAAAACCTGC
JA179 PBA1 2-17Δ (Cas9 repair)	ATATCATCGCACTACAGTAAAATTTTCATTTATAGCGATGCTCCCAGAGATTTCAAAAAACCTGCAATCTTTAGAGTCT
JA218 PBA1 F139A (gRNA F)	GACGTTATTATTTTCTATGGgtttt
JA219 PBA1 F139A (gRNA R)	CCATAGAAAATAATAACGTCgatca
JA220 PBA1 F139A (Cas9 repair)	TTCATTTGGAAAGACGTTATTATTTTCTATGGAaGAGAATgctATTAGTATATCGCCGATTTTTGGTAACATGATAAGTA
JA221 PBA1 D172A (gRNA F)	TCCCCTGATATAATAGTAATgtttt
JA222 PBA1 D172A (gRNA F)	ATTACTATTATATCAGGGGAgatca
JA223 PBA1 D172A (Cas9 repair)	AGCCCAGTTCTCCCCaGAcATtATAGTtATTGGCACCTCTGcTAAAATCGCCAGCATGAAGGTAATGACGGAAAATGAAT

Abbreviations: F, forward; gRNA, guide RNA; R, reverse.

Whole-cell lysates of logarithmic-phase cultures were prepared by a lithium acetate/sodium hydroxide method as previously described (26, 27). Lysates were analyzed by standard SDS-PAGE followed by standard immunoblotting. The following antibodies were used: anti-Pba1/2 (12), anti-alpha5 (14), and anti-Pgk1 (Invitrogen; catalog no.: 459250)

### Phenotypic analysis

Cells from overnight cultures were normalized by absorbance and spotted in threefold serial dilutions onto plates lacking or containing cadmium chloride (30 μM; Sigma; catalog no.: 202908) and cultured at 30 °C for the indicated number of days.

### Proteasome purification

CP was purified *via* a genomically integrated Pre1-TEV-ProA tag using immunoglobin G affinity chromatography as previously described (14). Note that high salt washes (500 mM NaCl) are used to separate CP from RP. Purified CP was analyzed by native gel electrophoresis followed by immunoblotting with the indicated antibodies.

### Structural analysis

The cryo-EM structure of Pre15S proteasome (Protein Data Bank code: 7LS6) has been previously described (14). Protein–protein interactions were annotated using PDBePISA (Table S1). Figures were prepared using both University of California San Francisco Chimera and ChimeraX.

### Sequence analysis of evolutionary conservation

ConSurf (https://consurf.tau.ac.il/) was used for the analysis of evolutionary conservation of Pba1 residues (28, 29).

## Data availability

Structures referenced here include the pre-15S proteasome (EMD-2350; Protein Data Bank codes: 7LS6) (14), 1RYP (30), 6FVY (6), and 4V7O (7).

## Supporting information

This article contains supporting information (28, 29).

## Conflict of interest

The authors declare that they have no conflicts of interest with the contents of this article.
