# Supplementary material for: Mechanism of proteasome gate modulation by assembly chaperones Pba1 and Pba2
Source: J Biol Chem. 2022 Apr 6;298(5):101906. doi: 10.1016/j.jbc.2022.101906 (PMC9065996; doi:10.1016/j.jbc.2022.101906)
Supplement: Supporting information [file mmc1.docx]

**Supporting Information**

TABLE S1. **Interaction interfaces between Pba1 and CP subunits.** Interactions were identified for the pre-15S structure (7LS6) using PDBePISA, and then manually annotated. :, indicates a sequence of contiguous residues.


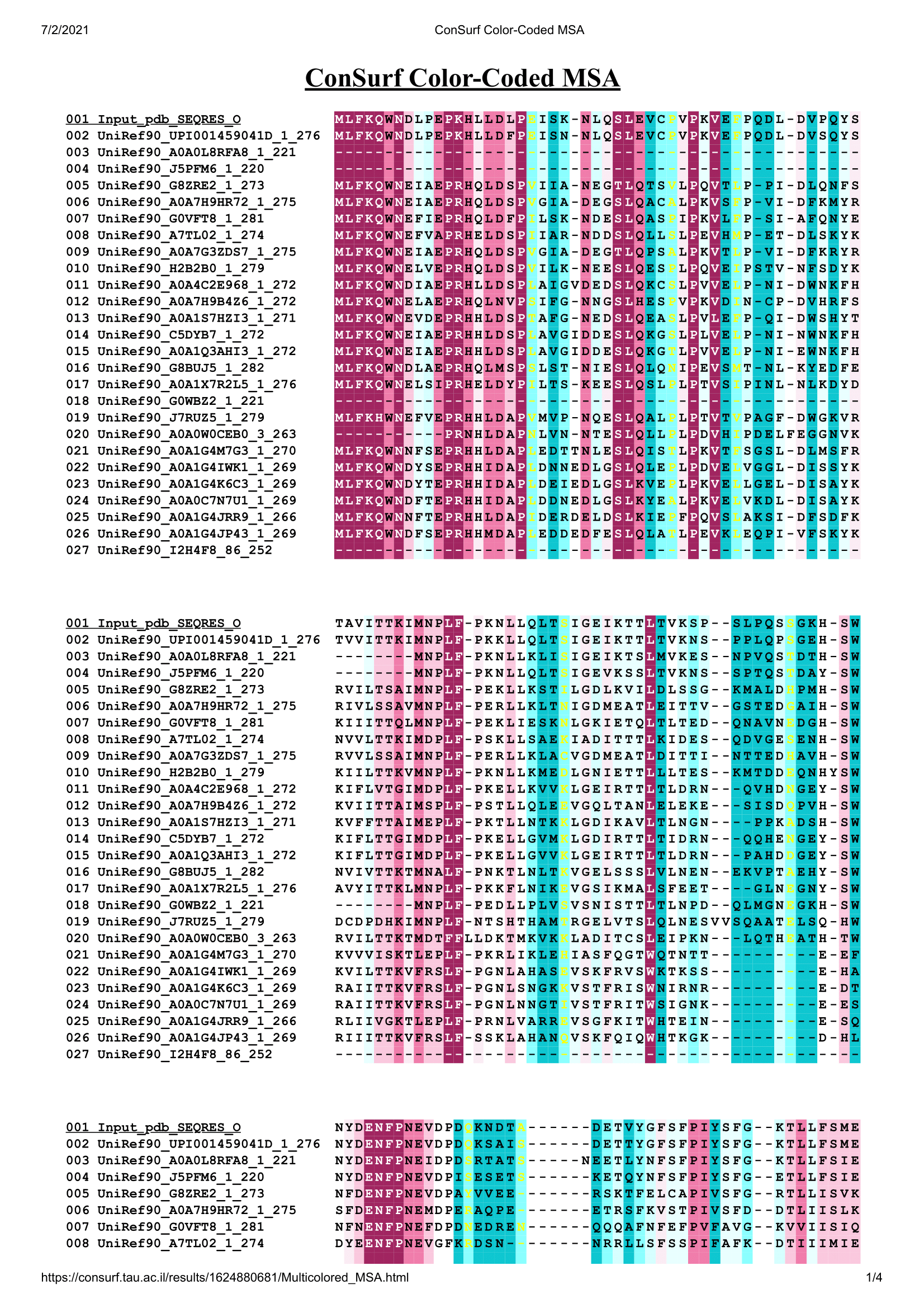


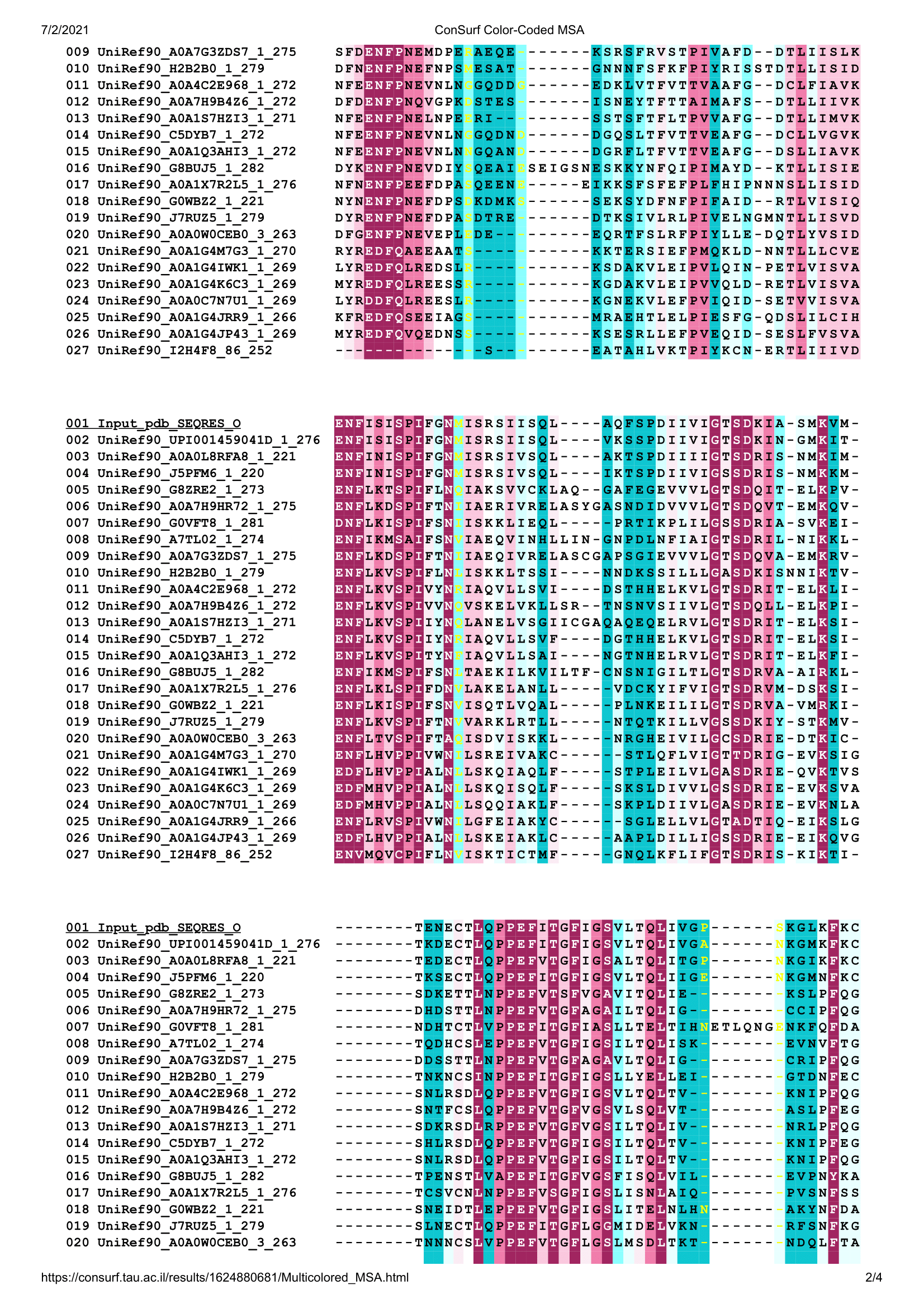


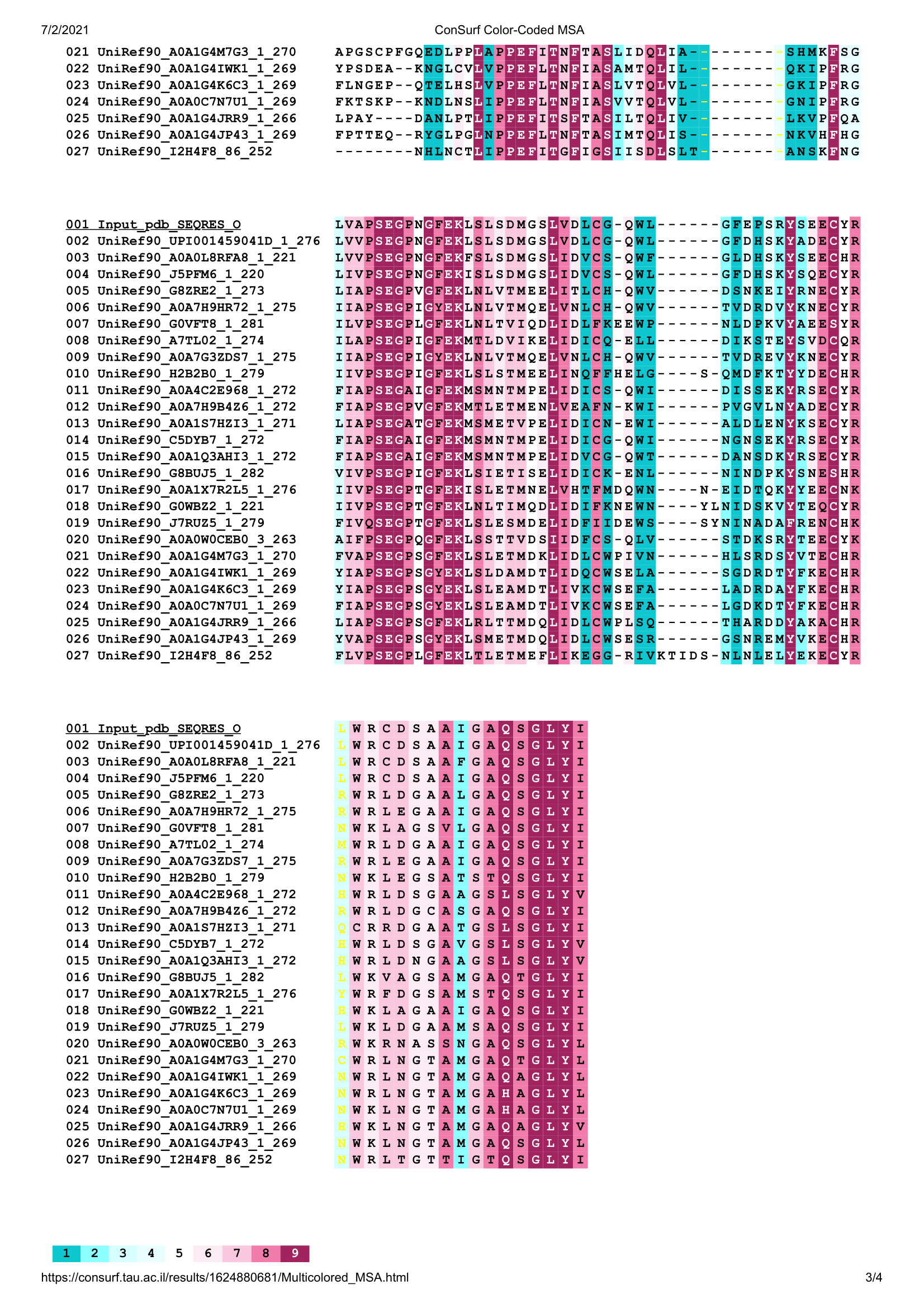

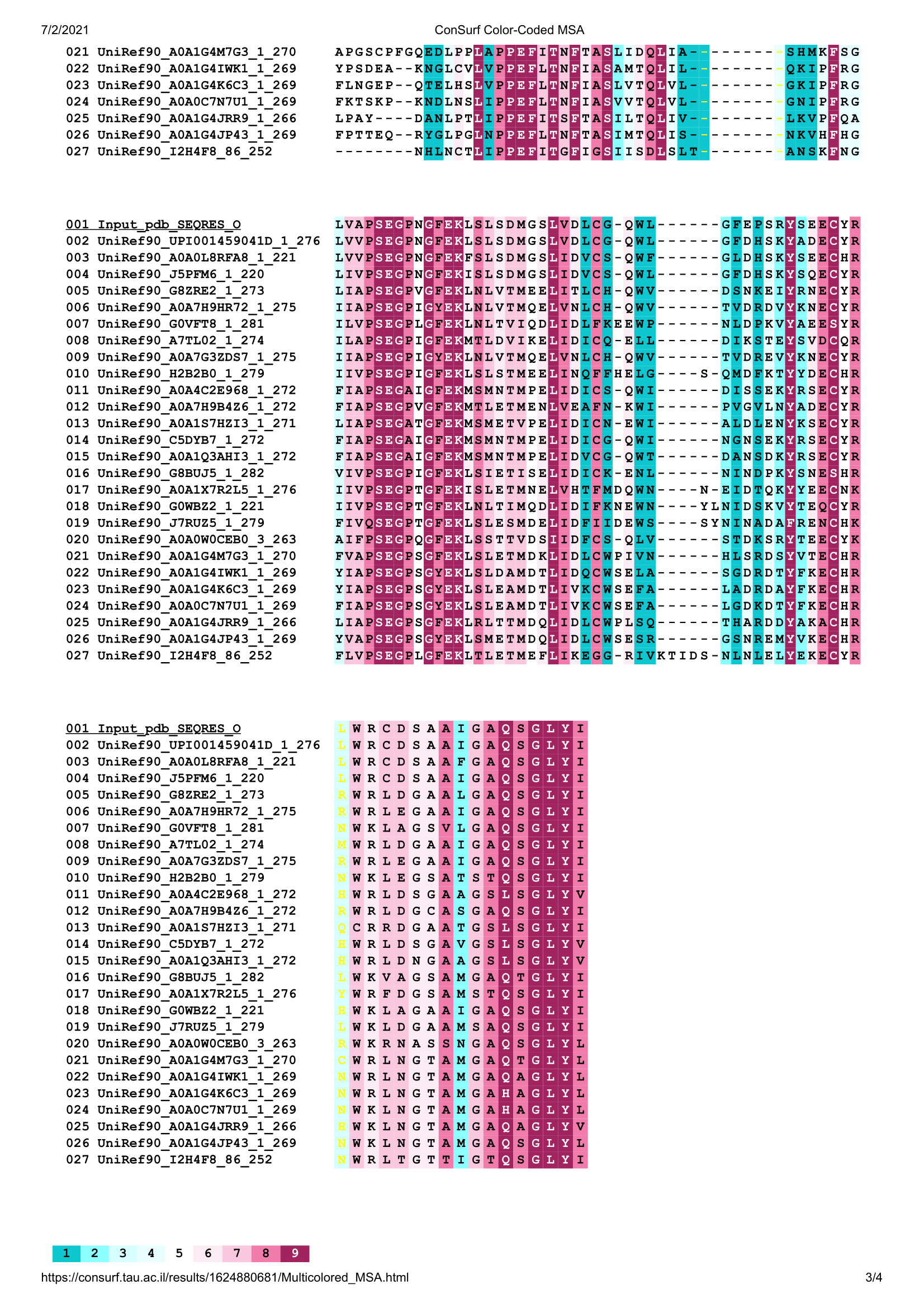

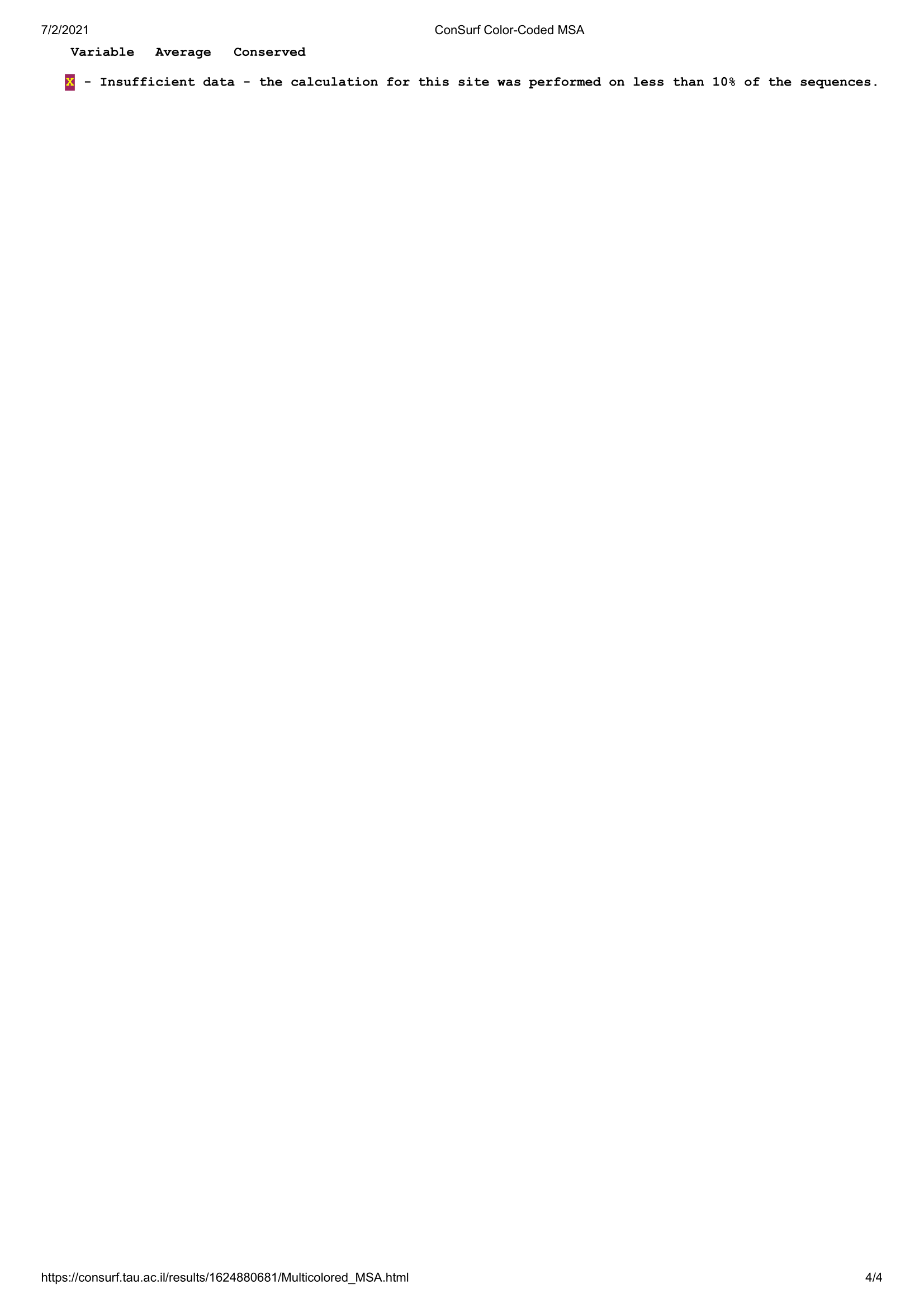


TABLE S2. **Evolutionary conservation of the key residues in Pba1.** Evolutionary sequence conservation was determined by the Consurf method (30, 31) which analyzes both the primary sequences and any relevant structures available in the Protein Data Bank (PDB). PDB 7LS6 was used as the input sequence/structure for this analysis. The putative Pba1 orthologs shown here are all from lower eukaryotes.

The specific species are as follows: 1. Saccharomyces cerevisiae (input); 2. Saccharomyces paradoxus; 3. Saccharomyces eubayanus; 4. Saccharomyces kudriavzevii; 5. Torulaspora delbrueckii; 6. Torulaspora globosa; 7. Naumovozyma castellii; 8. Vanderwaltozyma polyspora; 9. Torulaspora sp. CBS 2947; 10. Kazachstania africana; 11. Zygosaccharomyces mellis; 12. Zygotorulaspora mrakii; 13. Zygosaccharomyces TaxID=4953; 14. Zygosaccharomyces rouxii; 15. Zygosaccharomyces rouxii TaxID=4956; 16. Tetrapisispora phaffii; 17. Kazachstania saulgeensis; 18. Naumovozyma dairenensis; 19. Kazachstania naganishii; 20. Candida glabrata; 21. Lachancea fermentati; 22. Lachancea meyersii; 23. Lachancea sp. CBS 6924; 24.Lachancea lanzarotensis; 25. Lachancea mirantina; 26. Lachancea nothofagi; 27. Tetrapisispora blattae. Note that the Consurf analysis shows some species lacking apparent homology to yeast Pba1's N-terminus. The significance of this finding is unclear, but it should be noted that direct study of these putative Pba1 orthologs has not been carried out and detailed analysis of the sequencing/annotation data could be helpful in this regard.
